# Altered expression of microRNA in the airway wall in chronic asthma: miR-126 as a potential therapeutic target

**DOI:** 10.1186/1471-2466-11-29

**Published:** 2011-05-23

**Authors:** Adam Collison, Cristan Herbert, Jessica S Siegle, Joerg Mattes, Paul S Foster, Rakesh K Kumar

**Affiliations:** 1 Centre for Asthma and Respiratory Disease, University of Newcastle and Hunter Medical Research Institute, Newcastle, Australia; 2 Inflammation and Infection Research Centre, University of New South Wales, Sydney, Australia

## Abstract

**Background:**

The role of microRNAs (miRNAs) in regulating gene expression is currently an area of intense interest. Relatively little is known, however, about the role of miRNAs in inflammatory and immunologically-driven disorders. In a mouse model, we have previously shown that miRNAs are potentially important therapeutic targets in allergic asthma, because inhibition of miR-126, one of a small subset of miRNAs upregulated in the airway wall, effectively suppressed Th2-driven airway inflammation and other features of asthma. In the present study, we extended investigation of the therapeutic potential of miRNA inhibition to our well-established model of chronic asthma.

**Methods:**

Female BALB/c mice were systemically sensitised with ovalbumin (OVA) and chronically challenged with low mass concentrations of aerosolised OVA for up to 6 weeks. Airway tissue was obtained by blunt dissection and RNA was isolated for miRNA profiling. On the basis of the results obtained, animals were subsequently treated with either an antagomir to miR-126 (ant-miR-126) or a scrambled control antagomir once weekly during the 6 weeks of chronic challenge, and the effects on airway inflammation and remodelling were assessed using established morphometric techniques.

**Results:**

Compared to naïve mice, there was selective upregulation of a modest number of miRNAs, notably miR-126, in the airway wall tissue of chronically challenged animals. The relative increase was maximal after 2 weeks of inhalational challenge and subsequently declined to baseline levels. Compared to treatment with the scrambled control, ant-miR-126 significantly reduced recruitment of intraepithelial eosinophils, but had no effect on the chronic inflammatory response, or on changes of airway remodelling.

**Conclusions:**

In this model of chronic asthma, there was an initial increase in expression of a small number of miRNAs in the airway wall, notably miR-126. However, this later declined to baseline levels, suggesting that sustained changes in miRNA may not be essential for perpetuation of chronic asthma. Moreover, inhibition of miR-126 by administration of an antagomir suppressed eosinophil recruitment into the airways but had no effect on chronic inflammation in the airway wall, or on changes of remodelling, suggesting that multiple miRNAs are likely to regulate the development of these lesions.

## Background

The role of non-coding RNA species in the regulation of mammalian gene expression is becoming increasingly apparent [1,2]. Among non-coding RNAs, the microRNAs (miRNAs) are of particular interest. These are small non-coding RNAs of approximately 17-24 nucleotides, each of which is predicted to regulate hundreds of genes (both coding and non-coding) by post-transcriptional (and possibly also translational) silencing. There is currently an intense focus on the role of miRNAs in a variety of human diseases, ranging from cardiovascular disorders to malignant neoplasms, with active investigation of the potential of inhibiting miRNAs as a novel approach to treatment [3,4].

The role of miRNAs in inflammatory and immunologically-driven disorders is slowly being elucidated [5,6]. Studies from our group [7] have identified miRNAs as potentially important therapeutic targets in allergic asthma. In a mouse model of acute allergic bronchopulmonary inflammation induced by intranasal challenge with house dust mite (HDM) extract, we demonstrated selective upregulation of a small subset of miRNAs in airway tissues. Furthermore, we showed that inhibition of microRNA-126 (miR-126) by delivery of an antagomir (a cholesterol-linked single-stranded anti-sense RNA that selectively binds to this miRNA) effectively suppressed Th2-driven airway inflammation, mucus hypersecretion and airway hyper-responsiveness [7].

We therefore sought to extend investigation of the therapeutic potential of miRNA inhibition in asthma to a study in our well-established model of chronic asthma based on long-term low-level challenge with ovalbumin (OVA) [8,9]. This more closely replicates several key features of this disease, including acute-on-chronic inflammation of the airway wall, subepithelial and epithelial changes of remodelling, airway-specific hyper-responsiveness, and a spatial distribution of lesions corresponding to that observed in human asthma [10]. In this report, we describe the time course of altered expression of miRNAs in the airway wall in our model of chronic asthma and assess the potential of using an antagomir to inhibit miR-126 (the most highly-upregulated miRNA) as a therapeutic intervention.

## Methods

### Mice, sensitisation and challenge

The protocols employed for sensitisation and inhalational challenge have previously been described [11]. Briefly, specific pathogen-free female BALB/c mice aged 7-8 weeks (Animal Resources Centre, Perth, Western Australia) were systemically sensitised by intraperitoneal injection of 50 μg of alum-precipitated chicken egg OVA (Grade V, ≥98% pure, Sigma Australia) 21 and 7 days before inhalational challenge, then exposed to aerosolised OVA in a whole body inhalation exposure chamber (Unifab Corporation, Kalamazoo, MI) [12]. Chronic low-level challenge involved exposure to ≈3 mg/m^3 ^aerosolised OVA for 30 minutes/day on 3 days/week for up to 6 weeks. Particle concentration within the chamber was continuously monitored using a DustTrak 8520 instrument (TSI, St Paul, MN). All experimental procedures complied with the requirements of the Animal Care and Ethics Committee of the University of New South Wales (reference numbers: 06/119B and 08/09B). Data were collected from 6 animals per group for miRNA profiling and 8 animals per group for treatment with antagomirs. Control groups included naïve mice and mice that were not sensitised but were challenged for 6 weeks with aerosolised OVA.

For miRNA profiling, chronic challenge was performed for 1, 2, 4 or 6 weeks. Animals were sacrificed 48 hours after the final challenge.

### Isolation of Proximal Airway Tissue

Airway tissue was isolated by blunt dissection, using two pairs of forceps to separate lung parenchyma from the larger airways and leaving several generations of airway attached to the trachea [13]. Airway tissue was frozen in liquid nitrogen until RNA extraction was performed.

### Isolation of mRNA and miRNA

For assessment of miRNA, total RNA was isolated from blunt dissected distal airway tissue of individual animals using the mirVana miRNA Isolation kit (Ambion). For assessment of mRNA, RNA was isolated using TriReagent (Sigma) and following DNase treatment (Turbo DNase, Ambion), samples were reverse transcribed into cDNA using Superscript III (Invitrogen).

### miRNA microarray

miRNAs were enriched with the Ambion flashPAGE system. The Ambion 1564V1 probeset was printed on microarray epoxy slides by the Australian Genome Research Facility, Parkville, Australia. miRNA was polyadenylated and labelled with Cy3 using the mirVana miRNA labelling kit and arrays were hybridised and washed as described by Ambion. Slides were scanned using a GenePix4000B (Molecular Devices) and GenePix 6.0 software was used to quantify raw signal intensities. Analysis of microarray data was conducted using Genespring GX 11 software (Agilent). Percentile shift normalisation (75^th ^percentile) was performed with subsequent fold change calculations conducted against mean normalised naïve expression levels.

### Quantitative RT-PCR

qRT-PCR for miRNA was performed using TaqMan Gene Expression Assays for the respective miRNA (Applied Biosystems). miRNA expression was normalised to sno202 RNA. qRT-PCR for mRNA expression used primers that were custom-designed in house. Reactions were performed using an ABI Prism 7700 Sequence Detector (Applied Biosystems). Amplified products were detected using SYBR green and expression was normalised to hypoxanthine-guanine phosphoribosyl transferase.

### Antagomirs

Target miRNA sequences were downloaded from miRBase, Faculty of Life Sciences, University of Manchester, UK (http://www.mirbase.org/). We ordered antagomirs from Dharmacon. The scrambled antagomir was nonspecific RNA VIII, blasted against the mouse genome. The sequence of ant-miR-126 was: 5' mG.*.mC.*.mA.mU.mU.mA.mU.mU.mA.mC.mU.mC.mA.mC.mG.mG.mU.mA.*.mC.*.mG.*.mA.*. 3' -Chl, where ''m'' were 2'-OMe modified phosphoramidites, ''*'' were phosphorothioate linkages, and ''-Chl'' was hydroxyprolinol-linked cholesterol. For antagomir therapy, chronic challenge was performed as above for 6 weeks. Mice received either the antagomir to miR-126 (ant-miR-126) or the control antagomir based on the scrambled sequence (ant-scrambled) once per week intranasally. For both antagomirs, the dose was 50 μg in 50 μL sterile saline (25 μL per nare) as previously described [7].

### Assessment of airway inflammation and remodelling

In the antagomir and control-treated mice, asthmatic inflammation and airway wall remodelling were quantified in longitudinally oriented sections of formalin-fixed, paraffin-embedded tracheas, or horizontally oriented sections from the mid-zone of the single lobed left lung, as previously described [8]. Assessment included numbers of intraepithelial eosinophils and of chronic inflammatory cells in the lamina propria of the airway, extent of subepithelial fibrosis and grading of mucous cell change. The validity and reliability of the morphometric techniques we employed have been established in previous reports [8,14].

### Immunostaining

Expression of eotaxin in the airway epithelium was demonstrated using a goat polyclonal antibody to a 19-amino acid peptide corresponding to an epitope at the carboxy terminus of mouse eotaxin (Santa Cruz Biotechnology, Santa Cruz, California) (*sc*-6182). Immunoperoxidase staining of formalin-fixed, paraffin-embedded sections was performed following antigen retrieval in 0.01M citrate buffer (pH 6.0), as previously described [15]. Intensity of immunoreactivity was semi-quantitatively scored as grade 0 = no staining, grade 1 = weak staining, grade 2 = moderate staining and grade 3 = strong staining.

### Statistical analysis

Results are presented as mean ± SEM, or as medians (interquartile range) for grading. Differences between groups were assessed using a one-way ANOVA or Kruskal-Wallis test, followed by a Newman-Keuls or Dunn's post test as appropriate. The software package GraphPad Prism 5.01 (GraphPad Software, San Diego, CA) was used for data analysis and preparation of graphs.

## Results and Discussion

### Altered expression of miRNAs in the airway wall in chronic asthma

Compared to naïve mice, there was selective upregulation of a modest number of miRNAs in the airway wall tissue of sensitised, chronically challenged animals. Only 11 miRNAs exhibited a 2-fold or greater increase: these were miR-126, -197, -341, -145, -30c, -23b, -199a, -29a, -129-3p, -16 and -495 (Table 1). The complete dataset is deposited at ArrayExpress (http://www.ebi.ac.uk/arrayexpress) (accession number E-MEXP-3118).

**Table 1:** Fold change in expression of miRNAs in airway wall tissues during chronic challenge

	*Week 1*	*Week 2*	*Week 4*	*Week 6*
miR-126	3.11	7.93	4.56	2.33
miR-197	*1.41*	2.54	1.55	1.25
miR-341	1.23	2.37	1.62	1.39
miR-145	1.92	2.36	1.62	1.04
miR-30c	1.63	2.26	1.55	1.18
miR-23b	1.2	2.12	1.43	*1.03*
miR-199a	*1.19*	2.11	1.38	*1.19*
miR-29a	*1.09*	2.06	1.42	1.09
miR-129-3p	1.56	2.04	1.5	1.31
miR-16	1.2	2.03	1.4	1.03
miR-495	1.27	2.02	1.36	1.14

Changes in relative expression of individual miRNAs relative to levels in naïve animals, as assessed by microarray profiling. All miRNAs with >2-fold upregulation at 2 weeks of challenge are listed. Downregulation at other time points is shown in *italics*.

**Figure 1:**
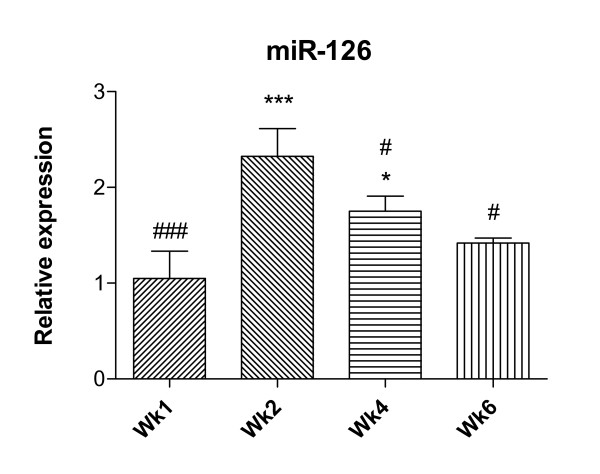
**Relative expression of miR-126**. qRT-PCR confirmation of upregulation of miR-126 in airway wall tissue of animals following chronic challenge. Significant differences compared to naïve controls are shown as * (p < 0.05), *** (p < 0.001); significant differences compared to mice challenged for 2 weeks are shown as # (p < 0.05), ### (p < 0.001)..

Notably, levels of expression of miR-126 were increased to a much greater extent than those of any other upregulated miRNA, and this was confirmed by qRT-PCR (Figure 1). There was clear evidence of regulation of changes in miRNA expression over time: the relative increase was maximal after 2 weeks of inhalational challenge and subsequently declined, so that except for miR-126 all had returned to baseline levels by 6 weeks (Table 1).

In the control group of animals that were not sensitised but were challenged for 6 weeks with aerosolised OVA, miR-126, -341 and -380-3p were found to be upregulated by 2-fold or greater. Of these, miR-126 was again the most highly upregulated, with a 5-fold increase compared to naïve mice. This is of interest because we have previously shown that although these animals are not systemically sensitised, there is low-level sensitisation via the respiratory tract as a consequence of the chronic challenge, leading to a specific humoral immune response [16].

Some of the current findings with respect to altered expression of miRNA in the airway wall are congruent with our previous report [7] which similarly detected upregulation of a limited number of miRNAs following induction of allergic inflammation [7]. In that previous study, which was based on a short-term model of HDM-induced asthmatic airway inflammation, miR-126 was the most highly upregulated miRNA in the airway wall. In the present study, we have confirmed that miR-126 is potentially a very important miRNA in asthmatic inflammation, because it was also the most highly upregulated miRNA in the chronic OVA challenge model. Interestingly, in the previous study miR-16 was also upregulated in the HDM model and this was again observed in the chronic OVA model. However, there were some important differences in the model of chronic asthma, notably the absence of demonstrable upregulation of miR-21, and evidence of upregulation of 10 additional miRNAs.

Because we assessed expression of miRNA in airway wall tissues, we do not have information about changes in specific cell types. Airway wall tissues include structural cells (such as airway epithelium, fibroblasts, smooth muscle, cartilage, vascular tissues) and recruited inflammatory cells. Importantly, unlike in short-term models of allergic inflammation of the airways, in our chronic challenge model there is a substantial change in the inflammatory cellular profile in the airway wall over time. Notably there is progressive accumulation of cells associated with chronic inflammation, especially CD3^+ ^T-lymphocytes and plasma cells [13,16]. Whether cell-specific changes account for the different profile of expression of miRNA in the chronic challenge model is at present unclear.

Whereas in the HDM model we assessed changes in expression of miRNA following two challenges 10 days apart, in the present study we were able to examine the time course of changes in expression of upregulated miRNAs. Remarkably, this revealed that upregulation was not sustained with continuing challenge, which could imply that expression of these miRNAs plays a more important role in the initiation rather than the perpetuation of asthmatic lesions.

### Effects of treatment with antagomir to miR-126 on changes of chronic asthma

**Figure 2:**
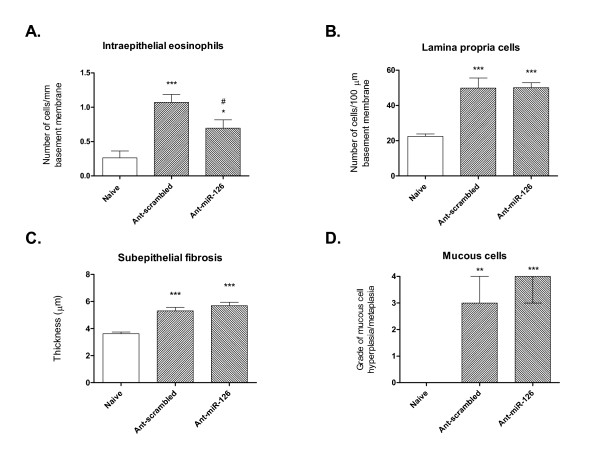
**Morphometric assessment of the effects of antagomir treatment**. Quantification of **(A) **profile density of intraepithelial eosinophils **(B) **profile density of chronic inflammatory cells in the lamina propria **(C) **thickness of subepithelial collagenisation (all assessed in the trachea) and **(D) **grade of mucous cell change (assessed in intrapulmonary airways) after 6 weeks of chronic challenge and antagomir treatment. Values are expressed as mean ± SEM (A-C) or median ± interquartile range (D); 8 animals were assessed per group. Significant differences compared to naïve controls are shown as * (p < 0.05), ** (p < 0.01), *** (p < 0.001); significant difference compared to mice treated with ant-scrambled is shown as # (p < 0.05).

Animals that were treated with the scrambled control antagomir developed airway wall changes that were indistinguishable from those observed in the chronic challenge model without any treatment [8], including recruitment of significant numbers of intraepithelial eosinophils, accumulation of chronic inflammatory cells in the lamina propria, subepithelial fibrosis and widespread goblet cell hyperplasia/metaplasia (Figure 2). Long-term administration of ant-miR-126 significantly reduced the numbers of intraepithelial eosinophils in the conducting airways (Figure 2A). However, treatment with ant-miR-126 had no effect on the chronic inflammatory response (Figure 2B). Similarly, changes of remodelling were essentially identical to those in mice treated with ant-scrambled (Figure 2C, D).

**Figure 3:**
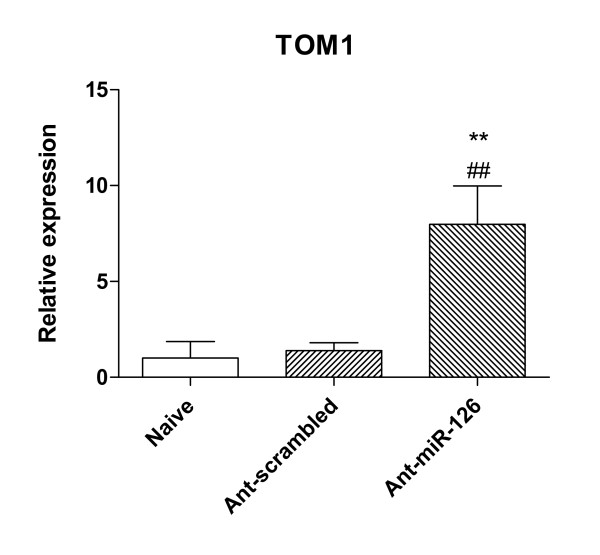
**Relative expression of mRNA for *TOM1***. qRT-PCR confirmation of upregulation of *TOM1 *in airway wall tissue of animals that received 6 weeks of chronic challenge and treatment with ant-miR-126. Significant difference compared to naïve controls is shown as ** (p < 0.01), compared to mice treated with ant-scrambled is shown as ## (p < 0.01).

To verify that delivery of ant-miR-126 was effective, we assessed the expression of TOM1 (target of Myb1) which is a negative regulator of IL-1β and TNF-α -induced signalling pathways. TOM1 has been defined as a target of miR-126 and is downregulated by it [17]. While there was no change in the expression of TOM1 in animals treated with ant-scrambled when compared to naïve mice, TOM1 was markedly and significantly upregulated in animals treated with ant-miR-126 (Figure 3). Whether TOM1 has any function in this model is unknown; however, this finding confirmed that ant-miR-126 was biologically active in the airway wall of these animals. Because samples from the antagomir-treated animals were processed for assessment of expression of mRNA, not miRNA, we were unable to directly confirm the effects of treatment with antagomirs on the levels of miR-126.

We have previously shown that accumulation of intraepithelial eosinophils is related to upregulation of expression of eotaxin in the airway epithelium in response to inhalational challenge [15]. To investigate whether the mechanism of the reduction in numbers of eosinophils in antagomir-treated mice was related to inhibition of expression of eotaxin, we performed immunostaining on sections of tracheas. As expected, there was increased immunoreactivity for eotaxin in the epithelium of animals treated with ant-scrambled (median grade 2.0, range 1-3). There was a reduction in animals treated with ant-miR-126 (median grade 1.0, range 0-2), although this difference was not statistically significant. Whether ant-miR-126 had any other cell-specific effects in this model is unknown.

In our previous study in an HDM-induced model of asthmatic airway inflammation, we showed that selective inhibition of miR-126 using a specific antagomir inhibited eosinophil recruitment and AHR [7]. The evidence that treatment with ant-miR-126 was also effective in suppressing eosinophil recruitment into the airways in the model of chronic asthma is encouraging in terms of the potential of antagomir therapy. However, in the short-term HDM model it was not possible to assess the effects of ant-miR-126 on chronic inflammation in the airway wall, or on the development of airway remodelling. Unfortunately, we found that treatment with ant-miR-126 did not inhibit the progression of these lesions. While the frequency of delivery of antagomirs was lower in the present study (once weekly rather than alternate days) it is not altogether surprising that targeting a single miRNA would not suppress all of the changes in a complex disorder such as chronic asthma. We have previously demonstrated that in many respects, the lesions of asthma are expressed differently in our chronic challenge model as compared to a short-term model of allergic pulmonary inflammation [9,18]. Thus our data suggest that ant-miR-126 alone has limited therapeutic potential in asthma. Nevertheless, they emphasise the importance of elucidating the role of other regulatory miRNAs in asthmatic airway inflammation, such as those we have identified in this study, which might allow the development of appropriate combination therapy.

## Conclusions

In this model of chronic asthma, inhalational challenge with OVA initially increased the expression of a small number of miRNAs in the airway wall, notably miR-126. By 6 weeks of challenge, however, this enhanced expression has largely declined to baseline levels, suggesting that sustained changes in miRNA may not be essential for perpetuation of chronic asthma. Inhibition of miR-126 by long-term administration of an antagomir suppressed eosinophil recruitment into the airways. However, this treatment had no effect on chronic inflammation in the airway wall, or on changes of remodelling, reinforcing the complexity of chronic asthma and the likelihood that multiple miRNAs regulate the development of these lesions.

## Competing interests

The authors declare that they have no competing interests.

## Authors' contributions

AC performed the miRNA studies under the supervision of JM. CH and JSS performed the animal experimental studies, immunostaining and morphometry. PSF and RKK conceived of the study and participated in its design and coordination. RKK and AC helped to draft the manuscript. All authors read and approved the final manuscript.

## Pre-publication history

The pre-publication history for this paper can be accessed here:

http://www.biomedcentral.com/1471-2466/11/29/prepub
